# Eco-friendly and ultra-sensitive electrochemical sensor for sertraline detection in pharmaceuticals and plasma

**DOI:** 10.1186/s13065-025-01602-2

**Published:** 2025-08-12

**Authors:** Ramy E. El-Bahnasawy, Hany A. Batakoushy, Hytham M. Ahmed

**Affiliations:** 1 Pharmaceutical Analysis Department, Faculty of Pharmacy, Menoufia University, Shebin Elkom, Menoufia 32511 Egypt; 2 Department of Pharmaceutical Analytical Chemistry, Faculty of Pharmacy, Menoufia National University, 70th km Cairo-Alexandria Agricultural Road, Menoufia, Egypt

**Keywords:** Sertraline, Glassy carbon electrode, Electrochemical polymerization, Methylene blue dye, Differential pulse voltammetry

## Abstract

In this work, methylene blue (MB) is electropolymerized to produce a modified glassy carbon electrode (GCE) that will enhance the electrochemical determination of sertraline (SRT), a selective serotonin reuptake inhibitor. The entire material characterization using Fourier transform infrared spectroscopy (FTIR), energy-dispersive X-ray analysis (EDX), and scanning electron microscopy (SEM) verified that the polymeric film of MB dye had successfully formed on the electrode surface. The modified electrode exhibited enhanced electron transfer kinetics, exhibiting ultra-sensitive SRT detection. According to ICH guidelines, Differential pulse voltammetry (DPV) has been used to estimate the limit of detection (LOD) for SRT, with concentrations ranging from 0.5 µM to 30.0 µM. The LOD value was calculated to be 0.28 µM. Voltammetric methods such as cyclic voltammetry, differential pulse voltammetry and electrochemical impedance spectroscopy were employed to investigate the electrooxidation behavior of SRT at various pH levels and scan rates. The results showed that poly methylene blue (PMB)/GCE exhibits improved electroactive surface area and enhanced charge transfer kinetics compared to bare GCE. High sensitivity, selectivity, and recovery percentage (99.08–101.09%) were demonstrated by the modified sensor (PMB/GCE) in detecting SRT in various matrices, including pharmaceutical samples and spiked plasma. The outstanding eco-friendliness and sustainability of the proposed method were thoroughly demonstrated using multiple assessment tools, including Analytical GREEnness (AGREE), AGREEprep, the Complex MoGAPI, and the newly developed Need, Quality, and Sustainability (NQS) index. In comparison to previously reported methods, the Red-Green-Blue (RGB) 12 algorithm proved to be more cost-effective, environmentally sustainable, analytically robust, and exhibited a higher degree of “whiteness.”

**Supplementary Information:**

The online version contains supplementary material available at 10.1186/s13065-025-01602-2.

## Introduction

Sertraline (SRT), an SSRI antidepressant, is widely used for its clinical efficacy. Its IUPAC name is *cis*-1 S, 4 S-N-methyl-4-(3,4-dichlorophenyl)-1, 2, 3, 4-tetrahydro-1-naphthalenaminehydrochloride, Fig. S1a [1, 2]. Depression has become one of the most pressing concerns affecting modern human societies, with significant social and economic consequences. Selective serotonin reuptake inhibitors (SSRIs), such as SRT, represent a first-line pharmacological treatment for anxiety and depressive disorders. SRT is also widely prescribed for the management of obsessive-compulsive disorder, post-traumatic stress disorder, premenstrual dysphoric disorder, panic disorder, and bipolar disorder. Sertraline concentrations in different biological samples must therefore be precisely determined using an analytical technique that is both sensitive and dependable [3, 4]. Although SRT has been quantified using methods such as Ultraperformance liquid chromatography UPLC [5, 6], High performance liquid chromatography HPLC [7–9], Gas chromatography– mass spectroscopy GC-MS [10, 11], Thin layer chromatography TLC [12], spectroscopic methods [13, 14], and some electrochemical approaches [15–20], have been used to quantify SRT. Because of their distinct benefits, modern electrochemical techniques are the specific focus of this study. Compared to conventional procedures, these technologies frequently require less sample preparation and produce less waste, and they have the potential to provide fast, sensitive, and economical analysis [21].

Electroanalytical techniques are widely used due to their ease of use, affordability, portability, and comparatively quick analysis periods. Electroanalytical methods have drawn a lot of interest recently for the identification of electroactive species. Among them, electrochemical techniques are particularly useful for applications in biological, environmental, agricultural, food, and pharmaceutical studies because of their great sensitivity and precision. For the detection of trace amounts of SRT, in complex matrices, such as pharmaceutical formulations and biological specimens, dependable electrochemical platforms must be developed. These techniques provide a productive and economical way to keep an eye on pollutants and active ingredients in ecological and clinical settings. However, bare electrodes face challenges like slow electron transport, reducing sensitivity. To overcome this, researchers modify electrodes with functional materials like electroactive dyes and nanomaterials, enhancing sensitivity and performance by reducing over potentials [22–31]. Furthermore, electrochemical sensors, leveraging these modified electrodes, represent a powerful class of analytical tools. They provide precise and reliable information about the identity and concentration of analytes, even at extremely low levels, making them valuable in a wide range of applications [32–41]. Electrochemical methods also present promising alternatives to sophisticated classical methods, offering the advantage of real-time analyte detection and providing insights into their electron-accepting or -donating properties. These techniques find widespread application in the quantification of a diverse array of organic and inorganic biomolecules [42–44].

The performance of these sensors, favored for their affordability, great sensitivity, quick response times, and straightforward instrumentation, is strongly influenced by the modified electrode materials. Thus, choosing the right electrode material is essential for the best electrochemical sensing results [45]. The objectives of this enhancement procedure are to increase resistance to fouling or contamination, increase selectivity, sensitivity, chemical and electrochemical stability, and expand the operating potential window [46, 47].

Electrochemical quantification of sertraline has been extensively studied in both pharmaceutical and biological samples, as documented in numerous reports. Various working electrodes, including bare Hanging mercury drop electrode (HMDE), and Carbon paste electrode (CPE) as well as modified electrodes, have been used to improve sertraline detection sensitivity (Table S1). MB, an electroactive dye, enhances sertraline’s electrochemical signal by mediating electron transfer between the drug and the electrode.

Electrochemical polymerization, also known as electropolymerization, is a commonly employed method for synthesizing coatings. This technique can be adapted for the incorporation of methylene blue (MB) on the surface of glassy carbon electrode (GCE). MB, a water-soluble cationic dye, excels as a redox mediator due to the presence of electron-rich sulfur and nitrogen atoms within its molecular structure [48] (Fig. S1b). MB is commonly employed in photocatalytic reactions due to its strong absorptivity and redox properties [49, 50]. A straightforward, repeatable technique that gives you exact control over film thickness is electropolymerization. This procedure involves immersing a working electrode in an electrolyte solution containing monomers, where polymerization is started by electrochemical oxidation. A conductive polymer film is produced by the reaction of radical cations produced by oxidation of MB. Compared to deposition procedures, this process has the advantages of stability, cost-effectiveness, and excellent electrical conductivity [51] (Scheme S1).

The mechanism of MB electropolymerization depends on factors like applied potential, electrolyte, and additives. MB can form a polymer, integrate into a co-polymer, or be deposited as a thin film. The resulting MB-modified electrode is useful for sensing, catalysis, and energy storage. By adjusting electropolymerization parameters, the film’s electrochemical properties and morphology can be tailored for specific applications [52, 53].

Being green because it offers a straightforward assessment of the approach by utilizing the twelve White Analytical Chemistry (WAC) principles, the level of technique sustainability will be determined using the whiteness meter [54–56]. Alternatively, procedures that allow for a global evaluation that takes into account all relevant factors should be chosen., such as those demonstrating respect for WAC principles, using (RGB12) algorithms. Because they earn the highest ranking in this total evaluation, the aforementioned procedures are consequently regarded as the highest quality. The recently developed (RGB) model [57–59], the color model in analytical chemistry extends green chemistry to red and blue. Red represents analytical efficiency (accuracy, precision, LOD), blue signifies economic and practical efficiency, and green reflects adherence to Green Analytical Chemistry (GAC). The eco-friendliness of the proposed technique was confirmed using AGREE, AGREE prep, Complex MoGAPI and (NQS index) assessments. This study aims to develop an electrochemical sensor based on a poly (MB) modified glassy carbon electrode (PMB/GCE) for the highly sensitive and selective determination of SRT.

## Experimental

### Apparatus

Electrochemical measurements were performed using an OrigaFlex OGF500 potentiostat voltammetric analyzer (OrigaLys, France) operated with OrigaMaster software. A conventional three-electrode setup was used, consisting of a silver/silver chloride (Ag/AgCl) reference electrode, a platinum wire auxiliary electrode, and a 3.0 mm glassy carbon working electrode. pH measurements were carried out using an Adwa pH-meter, Model AD1030 (Adwa Instruments, Romania). Scanning electron microscopy (SEM) and energy-dispersive X-ray spectroscopy (EDX) analyses were performed using a Quanta FEG 250 SEM (Thermo Fisher Scientific/FEI, USA). Fourier transform infrared (FTIR) spectra were recorded using a Jasco FT/IR–4600 spectrometer (Jasco, Japan).

### Chemicals and reagents

The used reagents and solvents were analytical grade-A. Sertraline (SRT) was kindly obtained from Pfizer Company, Egypt. Moodapex^®^ tablets (B.no., AT231282) was purchased from Apex Pharma (New Cairo, Egypt). Methylene blue powder (extra pure) was supplied by Alpha Chemika, India. Potassium ferricyanide K_3_Fe(CN)_6_ and potassium ferrocyanide trihydrate K_3_Fe(CN)_6_.3H_2_O were purchased from SMART-LAB, Indonesia. Potassium chloride (pure ≥ 99.0%) was purchased from Alfa Chemical, Egypt. Potassium di-hydrogen phosphate (assay 99%) and Di-sodium hydrogen phosphate was purchased from GEO specialty chemicals (Ambler, Pennsylvania, USA). Sodium chloride was supplied from El Nasr Pharmaceutical Chemicals Co, Egypt. Britton Robinson (BR) buffer solutions, with a pH range of 8.0 to 12.0, were the supporting electrolyte which was utilized for optimal circumstances. The components of BR buffer were 0.04 M of each (boric acid pure (assay 99.9%), acetic acid glacial and ortho phosphoric acid (extra pure 95%) were purchased from PioChem (6th of October City, Giza, Egypt) and Loba Chemie Pvt. Ltd. (Navi Mumbai, Maharashtra, India). 0.2 M sodium hydroxide was used to bring the buffer solution’s pH to the required level. All the prepared solutions were dissolved in distilled water.

### Preparation of standard solution

To prepare stock solutions, accurately weigh 15.30 mg of standard drug was transferred into 50 mL volumetric flasks. The drug was dissolved in distilled water after sonicating for 30 min. achieving a final concentration of 1.0 mM SRT. Working standard solutions were then prepared by further diluting suitable volumes of the stock solutions using B.R. buffer maintained at a pH of 9.0. The same procedures are used for the simultaneous determination of the stock solutions of ascorbic acid (AA) and uric acid (UA). 8.4 mg of UA are dissolved in 50 mL of distilled water to generate a 1 mM stock solution. After that sonicated for 40 min. 8.8 mg of AA were dissolved in 50 mL of distilled water resulting in a 1 mM stock solution.

### Sample preparation

#### Pharmaceutical sample preparation

Five tablets’ contents were precisely weighed, ground into a fine powder, and combined in a mortar and pestle. 50 mL of ethanol (purity; 99.9%) was used to dissolve 15.30 mg of the powdered combination, which is equal to 1.0 mM of SRT. The resultant solution was sonicated for 15 min to guarantee total dissolution. A non-sterile filter syringe (0.22 μm) was then used to filter the solution.

#### Spiked plasma sample preparation

Drug free human blood sample was collected from healthy non-smoking volunteer. The blood sample was centrifuged at 2,000 rpm for 15 min. one milliliter of the supernatant was mixed with 2 mL of acetonitrile and centrifuged at 6,000 rpm for 20 min in order to denature the protein. Therefore, 40 µL of the separated plasma was injected into different volumes of SRT standard solution (1 × 10^− 3^ mol L^− 1^) containing BR buffer (pH 9.0).

### Development of modified GC/ PMB

**Fig. 1:**
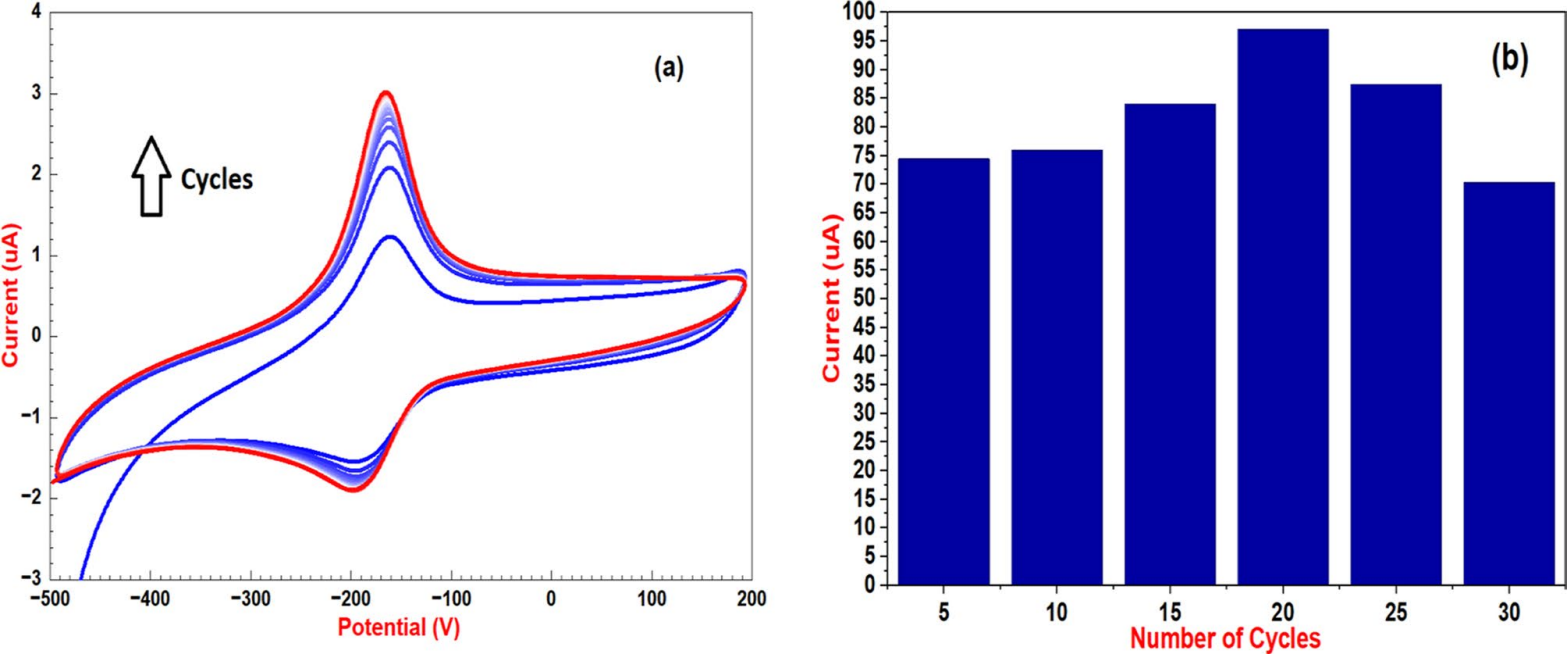
(**a**) cyclic voltammetry of MB polymerization on GCE and (**b**) Effect of cycles polymerization on GCE

In order to perform the electropolymerization procedure, GCE was submerged in a pH 7.0 solution that contained 1.0 mM MB and 0.1 M Phosphate buffer saline (PBS). The potential range used to measure cyclic voltammetry was − 0.5 V to 0.2 V with scan rate 0.05 V/S [60–62]. (Fig. 1a). Adsorption of MB monomers onto the electrode surface is the first step in the electropolymerization of MB on a glassy carbon electrode. In the initial cyclic voltammetry scan, a sharp rise in anodic current that indicates the onset of polymer formation. This process generates reactive cation-radical species. Anodic and cathodic peak currents both increase in strength with subsequent voltammetric cycles, and their corresponding potentials move toward greater positive and negative values. This behavior suggests the progressive growth of the polymer film on the electrode surface. Furthermore, increasing the scan rate accelerates the deposition process, resulting in a thicker polymer coating. The dynamic character of electropolymerization and its impact on film thickness are highlighted by this observation [60]. By adjusting the number of voltammetric cycles within a range of 5 to 30 cycles, the thickness of the deposited MB polymer film can be effectively controlled. Therefore, 20 cycles for electropolymerization have been selected as the investigative parameter for electrochemical studies of SRT (Fig. 1b).

### Electrochemical behavior

To evaluate the analytical capabilities of the fabricated sensor, the PMB/GCE electrode was used to investigate the electrooxidation of SRT. This involved CV, DPV and EIS studies at varying pH and scan rates. The performance of the electrode was further assessed through DPV analysis of SRT under standard conditions and in real samples.

## Results and discussion

### Characterization

#### Surface morphology of polymeric film

**Fig. 2:**
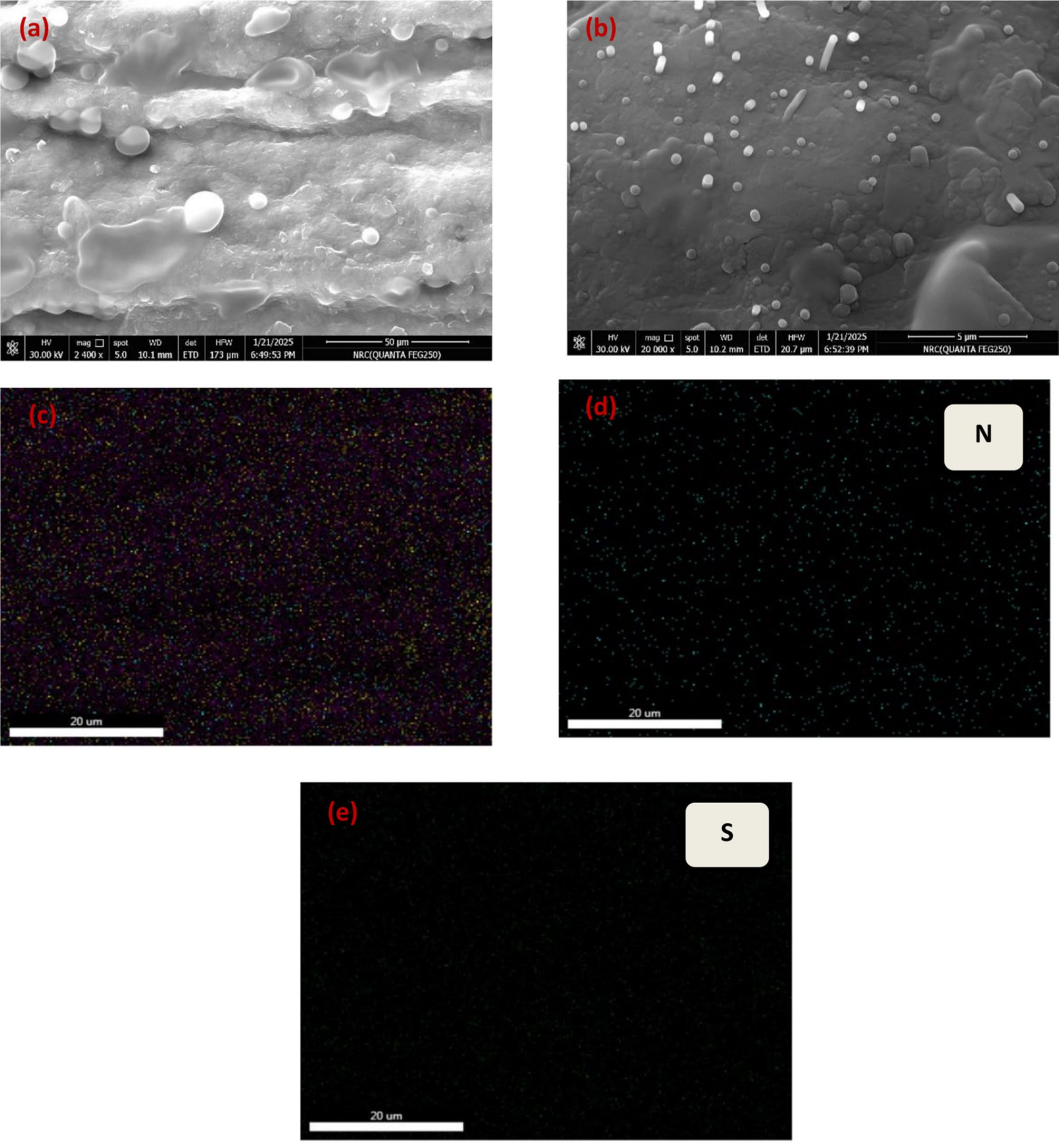
(**a**;**b**) SEM images of polymeric film, EDX mapping (**c**) uniform distribution of all atoms, (**d**) nitrogen atoms distribution and (**e**) sulfur atoms distribution

SEM and EDX mapping studies have been used to evaluate the surface morphology of PMB/GCE. Microscopic observations revealed that the PMB-modified GCE exhibited a unique morphology for granular surface with the presence of numerous small, round or slightly elongated protrusions scattered across the surface having a slightly grainy texture, contributing to an increase in the electrode’s surface area (Fig. 2a and b). To further characterize these morphological alterations, EDX analysis was performed, EDX spectrum of the PMB/GCE electrode revealed two additional low-intensity signals (N and S) to the existed signals of carbon and oxygen. (Figure 2c, d and e). The effective deposition of the PMB film onto the GCE surface is confirmed by these signals, which are typical of CH = S + and N = CH bonds.

To confirm the outputs, the chemical composition of the formed polymer was studied using FTIR in the 400–4000 cm^− 1^ range relating to the basic methylene blue powder and the outcomes were presented in (Fig. S2) A broad band located around 3400 cm^-1^ is often associated with O-H stretching vibrations. Formation of O-H group is suggesting oxidation of monomer during electrochemical polymerization. A sharp band at 2900–2800 cm^-1^ is related to C-H stretching vibrations present in both spectra. Their intensities and positions might differ due to changes in the molecular environment of the methylene blue upon polymerization. An additional peak observed at 2350 cm^-1^ in the spectrum of the modified electrode, which is absent in the spectrum of the methylene blue powder, suggests the formation of a C = O group, possibly due to oxidation. At 1600–1500 cm^-1^, this region is crucial for identifying aromatic ring vibrations and C = N stretching in methylene blue. The blue spectrum might show shifts or changes in the intensity of these peaks, indicating alterations in the electronic structure of the methylene blue molecule during polymerization. At 1300–800 cm^-1^, this region often shows peaks related to C-N stretching and C-S stretching. The two spectra can reveal changes in the chemical environment of these functional groups.

#### Electrochemical characterization of PMB/GCE

Cyclic voltammetry of potassium ferrocyanide system; a 5 mM K_4_Fe(CN)_6_/K_3_Fe(CN)_6_ redox probe in 0.1 M KNO_3_ electrolyte revealed a significant increase in peak current for the PMB/GCE compared to the bare GCE. This observation indicates an increase in the electroactive surface area of the modified electrode and suggests enhanced electrocatalytic activity. The total electroactive surface area of the modified glassy carbon electrode was determined using the Randles-Sevcik equation.

$${I_{pa}} = {\rm{ }}\left( {2.69{\rm{ }} \times {{10}^5}} \right){n^{3/2}}{A_0}{C_0}{D_0}^{1/2}{\upsilon ^{1/2}}$$

The anodic peak current (Ipa), the number of electrons transferred (n), electroactive surface area (A_0_), bulk concentration of the analyte (C_0_), diffusion coefficient (D_0_), and scan rate (ʋ). Electroactive surface areas of 17.85 cm² for the bare GCE and 19.03 cm² for the PMB/GCE were found by analyzing linear plots of peak current against the square root of scan rate. Significant increase in the electroactive surface area of the modified electrode demonstrated. The modified electrode exhibits a substantial increase in electroactive area, demonstrating significantly improved electrochemical performance and enhanced sensitivity for analytical applications.

#### Electrochemical impedance spectroscopy (EIS)

Electrochemical Impedance Spectroscopy (EIS) was utilized to examine the interfacial properties of the electrode surfaces. Impedance, representing the overall opposition to alternating current flow at a given frequency, was measured. Nyquist plots, depicting the relationship between the real (Z’) on x-axis and imaginary (Z”) components of impedance on y-axis, were generated to analyze diffusion-limited processes (Fig. S3). The study utilized redox couple (5mM potassium ferro/ferricyanide) in 0.1 M potassium chloride with two electrodes: bare GCE and PMB/GCE. EIS measurements were conducted at amplitude of 5 mV and a frequency range of 100 kHz to 100 mHz. To further understand the electrochemical behavior, the experimental EIS data was fitted to a modified Randles equivalent circuit, comprising solution resistance (Rs), charge transfer resistance (Rct), film resistance (Rf), double-layer capacitance (Cdl), film capacitance (Cf), and Warburg impedance (Zd). Analysis revealed a significantly lower Rct value (0.124 kΩ) for PMB/GCE compared to bare GCE (1.348 kΩ), indicating enhanced charge transfer kinetics at the modified electrode. The modified electrode demonstrated superior conductivity, facilitating faster reaction kinetics at its surface.

#### UV-Vis spectroscopy

Ultra-violet spectroscopy (UV-Vis) was employed to characterize the individual components (SRT and MB) and their mixture. Fig. S4 illustrates the absorbance spectra obtained in the wavelength range of 230–730 nm. SRT showed prominent absorption peaks at (273 nm) in the UV region, which can be attributed to delocalized electrons of aromatic rings that exhibits π-π* transition. The spectrum of MB exhibited a characteristic absorption peak at 660 nm, corresponding to the π-π* transition that responsible for its blue color. MB has an extensive conjugated system of alternating single and double bonds. The UV-Vis spectrum of the mixture of MB and SRT exhibits two distinct absorption regions. A prominent, high-intensity peak is observed in the visible region at 660 nm, corresponding to the characteristic absorption of MB. In the UV region, a significantly lower intensity peak is observed at 273 nm, refers to the absorption by SRT.

### Effect of pH

**Fig. 3:**
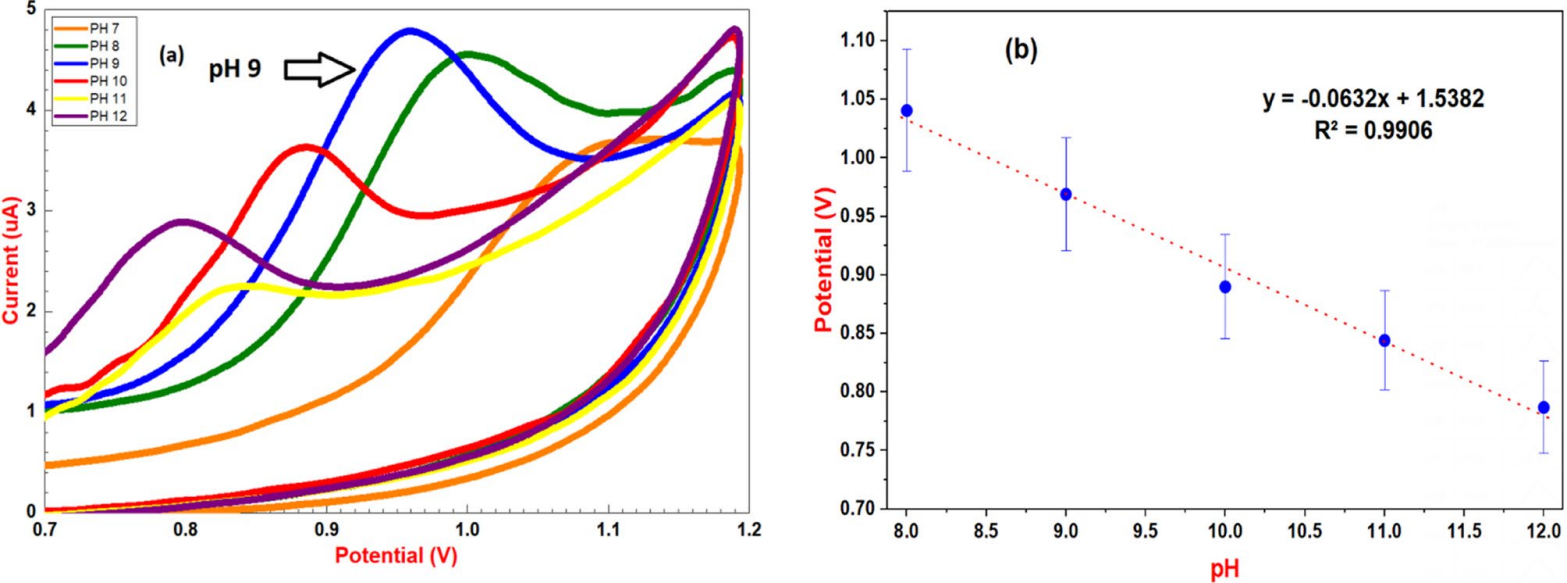
(**a**) Cyclic voltammetric responses of 1.0 × 10^− 3^ mol L^− 1^ SRT at different pH values (8.0–12.0) using PMB/GCE using scan rate of 0.1 V s^− 1^ and (**b**) The inset displays the linear relation of potential (V) versus pH

pH significantly affects sertraline’s electrochemical behavior, oxidation mechanism, solubility, stability, and analyte species. To investigate this pH influence, the voltammetric measurements were conducted using 0.04 M BR buffer at different pH values. At slightly acidic pH values (5–7), sertraline showed minimal electrochemical activity. No distinct peaks were observed at pH 5 and 6, while a small, poorly defined peak appeared at pH 7, indicating limited electrochemical response under these conditions (Fig. S9). In contrast, within the alkaline pH range (8–12), clear electrochemical signals were observed. This approach aims to optimize the experimental conditions by examining how pH variations affect the analyte’s electrochemical behavior within potential window from 0 to 1.2 V and scan rate of 0.1 V/s (Fig. 3a). The oxidation of SRT at PMB/GCE exhibits pH dependence, with the peak potential shifting negatively as pH increases, indicating proton consumption during the oxidation reaction. Consequently, B-R buffer at pH 9.0 was chosen as the appropriate pH for further investigations. The peak potential (Ep) showed a linear dependence on pH (R² = 0.9906) (Fig. 3b), fitting the equation: Ep (V) =– 0.0632 pH + 1.5382 V. The observed slope of 0.063 V/pH demonstrates excellent agreement with the Nernst prediction of 0.059 V/pH. The total number of electrons involved in the electrochemical oxidation of SRT was estimated using this equation, expressed as: $\:\text{S}\text{l}\text{o}\text{p}\text{e}=0.0591\:\text{x}\:\left(\frac{\text{m}}{\text{n}}\right)$

where m is the number of protons and n is the number of electrons involved in the reaction. By incorporating the experimentally observed slope from the plot of peak potential (Ep) versus (pH), the calculated m/n ratio was ~ 1.06, indicating a strong correlation and suggesting a proton-electron stoichiometry close to 1:1 in the SRT electro-oxidation process [63].

### Effect of scan rate

Voltammetric studies often utilize scan rate as a key parameter to investigate reaction kinetics, physicochemical properties, and the number of electrons involved in the electrochemical process. In this study, the influence of scan rate (ranging from 0.025 to 0.2 V/s) was examined within potential window from 0 to 1.2 V to determine the extent of diffusion control or mass transfer limitations on the electrode reaction of 1.0 mM SRT in 0.04 M BR at pH 9.0 (Fig. S5a). To investigate the reaction mechanism, the relationship between peak current (Ipa) and both scan rate (υ) and its square root (υ^1/2^) was analyzed. Fig. S5b demonstrates a linear correlation between Ipa and υ^1/2^, represented by the equation Ipa (µA) = 1.1337υ (V/s) + 0.5161 with an R^2^ of 0.9855. This linear relationship strongly suggests that the oxidation process of SRT at the PMB/CPE electrode is diffusion-controlled. Fig. S5c demonstrates linear regression analysis of the relationship between anodic peak potential (Epa) and the logarithm of scan rate (log ν) was employed to determine key electrochemical parameters Epa = 0.0874 log υ (V/s) + 1.113 with R^2^ = 0.9947. Specifically, the electron-transfer rate constant (ks) of the PMB/GCE electrode system was calculated using Laviron’s theory. The charge transfer coefficient (α), a crucial parameter in understanding electrode reaction kinetics, was also determined using the derived equations.

E = E^o^ +$\:\:\left(\frac{2.303\:\text{R}\text{T}}{\text{a}\text{n}\text{F}}\right)$ log$\:\:\left(\frac{\text{R}\text{T}\text{K}^\circ\:}{\text{a}\text{n}\text{F}}\right)$ +$\:\:\left(\frac{2.303\text{R}\text{T}}{\text{a}\text{n}\text{F}}\right)$ log υ. The α and ks was calculated to be 0.34 s^− 1^ and 1.75 s^− 1^ respectively.

### Oxidation mechanism of SRT

Electrochemical studies at (PMB/GCE) revealed that the irreversible oxidation of SRT is governed by a mixed diffusion and adsorption process. The two-electron, two-proton oxidation mechanism of SRT has been previously reported [18, 64] (Scheme S2).

### Optimization of the developed analytical technique

#### Calibration curve and linearity range

**Fig. 4:**
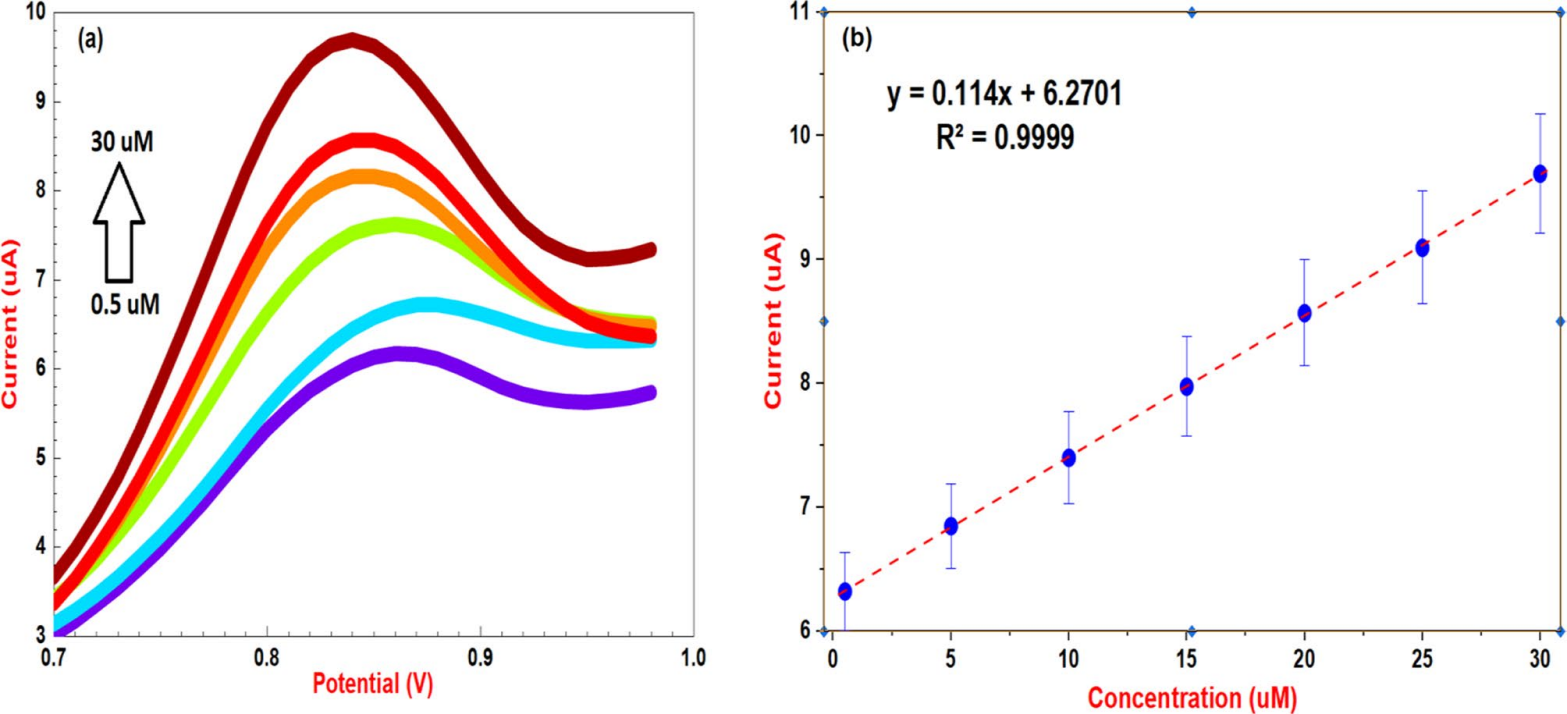
(**a**) Differential pulse voltammetric responses using PMB/GCE in BR buffer pH 9.0 at scan rate 0.1 V/S at various concentration (0.5–30.0 µM), (**b**) Calibration plot of the oxidation peak current versus the concentration range of SRT

The analytical capabilities of a PMB/CPE modified electrode for the electrochemical determination of SRT were thoroughly investigated in accordance with ICH guidelines [65]. Differential pulse voltammetry (DPV) was chosen as the analytical method due to its high sensitivity within potential window from 0 to 1 V with scan rate at 0.1 V/S, Pulse amplitude 0.15 V, step potential 0.001 V and Pulse width / time 0.020 s. Optimization of the supporting electrolyte revealed that a 0.04 M BR buffer at pH 9.0 provided the best performance, with a well-defined SRT oxidation peak observed at 0.86 V (Fig. 4a). A linear calibration curve was obtained across the concentration range of 0.5 to 30 µM, described by the equation Ip (µA) = 0.114 C + 6.2701 (r² = 0.9999) (Fig. 4b). By calculating analytical detection limits, the sensitivity of the approach was assessed. The following formulas were used to determine the limit of detection (LOD) and quantification (LOQ) in compliance with ICH guidelines.

$$LOD{\rm{ }} = {\rm{ }}3.3(\sigma /S),{\rm{ }}LOQ{\rm{ }} = {\rm{ }}10(\sigma /S)$$

Where σ represents the standard deviation of the response and S denotes the slope of the calibration curve. LOD and LOQ were calculated to be 0.286 µM and 0.868 µM, respectively, demonstrating the method’s high sensitivity. Concentrations above 30 µM resulted in a loss of linearity, likely due to surface saturation of the electrode. The validated method for sertraline analysis is simple, rapid, cost-effective, and environmentally friendly compared to conventional chromatographic and spectroscopic techniques. Validation parameters are in Table 1.

**Table 1:** Statistical parameters of validation for the performance of PMB/GCE in determination of SRT

Parameter	Result
Linearity range	0.5 − 30 µM
Regression coefficient (r^2^)	0.9999
Slope	0.114
Intercept	6.2701
Standard deviation of slope (S_b_)	0.0006
Standard deviation of intercept (S_a_)	0.0099
Limited of detection (LOD)	0.2867 µM
Limit of quantification (LOQ)	0.8688 µM

#### Accuracy

Accuracy was assessed using the PMB/GCE platform. Recovery studies used five SRT concentrations (5.0, 10.0, 15.0, 20, and 25 µM). These concentrations covered the full analytical range. Recoveries were excellent, from 99.08 to 101.09% as shown in (Table 2).

**Table 2:** Accuracy and intermediate precision of PMB/GCE for SRT determination

Sample number	Taken conc. (µM)	Found conc. (µM)	% Recovery ± RSD
Accuracy			
(1)	**5.0**	5.0547	101.0936 ± 0.0070
(2)	**10.0**	9.9290	99.2895 ± 0.0027
(3)	**15.0**	14.9670	99.7797 ± 0.0030
(4)	**20.0**	20.1950	100.9751 ± 0.0030
(5)	**25.0**	24.7711	99.0842 ± 0.0100
Intra-day precision	**5.0**	5.0517	101.0351 ± 0.0080
	**10.0**	9.9699	99.6988 ± 0.0047
	**15.0**	14.99327	99.9552 ± 0.0012
Inter-day precision	**5.0**	5.0137	100.2749 ± 0.0158
	**10.0**	9.9670	99.6696 ± 0.0123
	**15.0**	14.9436	99.6238 ± 0.0101

RSD: Relative Standard Deviation

#### Precision

Using DPV, precision was carefully examined. Intermediate precision and repeatability were assessed. Intra-day precision was determined from nine readings of three concentrations 5.0, 10, and 15 µM. Inter-day precision testing on three other successive days (Table 2). It was noted that RSDs for both were less than 2%, which indicated to the proposed method is a precise.

### Application of the modified electrode (PMB/GCE)

#### Pharmaceutical sample analysis

The fabricated PMB/GCE sensor was used to measure SRT directly in dissolved Moodapex^®^ tablets (50 mg SRT/tablet). This approach avoided complex sample preparation, proving its practicality for routine pharmaceutical analysis. Excellent recovery rates (Table 3) confirmed the method’s accuracy and selectivity, even with the presence of other inactive ingredients in the tablets. This superior selectivity is due to the unique interaction between SRT and the MB polymer on the electrode surface, allowing for direct analysis without formulation components interfering.

**Table 3:** Application of PMB/GCE to pharmaceutical tablets

Sample	Technique	Taken conc. (µM)	Found Conc. (µM)	% Recovery
Tablets	**DPV**	**10.0**	10.0277	100.2770 ± 0.0012
		**20.0**	19.6399	98.1995 ± 0.0016
		**30.0**	30.1662	100.5540 ± 0.0013

#### Spiked plasma sample analysis

To assess the method’s potential for therapeutic drug monitoring, we measured SRT in human plasma samples. Measuring SRT levels in blood plasma is difficult because peak plasma concentrations (Cmax) typically reach 20–55 µg/L within 4–8 h. These clinically relevant concentrations are below the measurable range of standard, unmodified GCE. This highlights the crucial role of modifying the electrode surface with the PMB. The increased sensitivity provided by this modification allowed us to directly measure SRT in plasma samples without the need for complex extraction procedures. Recovery studies (Table 4), performed on spiked plasma samples, the results of which are demonstrated the method’s ability to accurately monitor drug levels in complex biological matrices like plasma. The absence of notable matrix effects, meaning interference from other components in the plasma, and The method’s suitability for application in clinical settings is confirmed by the constant recovery values. The polymer’s unique electron transport properties and resistance to fouling enable reliable plasma sample analysis, minimizing interference and reducing the requirement for thorough sample cleaning.

**Table 4:** Application of PMB/GCE to spiked plasma

Sample	Technique	Taken conc. (µM)	Found Conc. (µM)	% Recovery
Spiked plasma	**DPV**	**15.0**	14.7416	98.2773 ± 0.0025
		**20.0**	20.1089	100.5445 ± 0.0013
		**25.0**	24.9823	99.9292 ± 0.0011

A calibration curve was constructed using five serotonin concentrations (10, 15, 20, 25, and 30 µM), each measured in triplicate. The average peak current values were plotted against concentration, yielding a linear regression equation Ip (µA) = 0.2094 C + 5.8031 with an excellent correlation coefficient R² = 0.9996. To evaluate the accuracy and applicability of the proposed sensor in real samples, recovery studies were conducted using the standard addition method. Known concentrations of serotonin (15, 20, and 25 µM) were spiked into a pre-analyzed real sample matrix. The recovery percentage was calculated using the formula:

$${\rm{Recovery }}\left( {\rm{\% }} \right){\rm{ = }}\left[ {{\rm{Found / True}}} \right]{\rm{ \times 100}}{\rm{.}}$$

The obtained recoveries ranged from 98.27 to 100.54%, indicating high accuracy and minimal matrix interference.

#### Selectivity study

The simultaneous determination of SRT with UA and AA is crucial for accurate clinical analysis. Although AA does not chemically interact with SRT, its acidic nature, when taken orally as vitamin C, can influence SRT excretion via renal competition. Fig. S6 displays the analysis of 0.05 mM SRT in the presence of 0.1 mM UA and 0.1 mM AA. Notably, the mixture at pH 9 exhibits only two distinct peaks, corresponding to SRT and UA. The absence of the AA peak in the mixture is likely due to its instability and degradation at pH 9, which is not optimal for AA’s stability. This observation underscores the importance of considering the pH-dependent behavior of analytes when developing methods for simultaneous determination in complex matrices. The successful separation of SRT and UA, despite the absence of AA signal, highlights the suitability of our PMB/GCE modified electrode for the reliable quantification of SRT in biological samples, even when pH conditions may compromise the stability of certain interferents.

### Comparison of the proposed sensor with the pervious methods

To benchmark the performance of the PMB/GCE sensor developed in this study, its key analytical characteristics were compared against previously reported electrodes used for SRT quantification (Table S1). The PMB/GCE sensor exhibits a superior detection limit, proficient all previous reports.

### Sustainability evaluation

#### The whiteness metric

The Red, Green, Blue (RGB12) tool [55], developed by Paweł Nowak and colleagues in June 2021, offers a straightforward assessment of WAC principles. The twelve algorithms within RGB12 are categorized into three main groups: reds, greens, and blues. The green subgroup (G1–G4) evaluates key GAC attributes such as toxicity, waste generation, energy consumption, and potential impacts on humans, animals, and genetically modified organisms. The red subgroup (R1–R4) focuses on validation measurements. The blue subgroup (B1–B4) addresses cost-effectiveness, time efficiency, and economic factors. The RGB algorithm calculates a “whiteness” value by summing the scores from the three groups, reflecting the methodology’s alignment with WAC criteria. As illustrated in Fig. S7, the proposed approach proves to be more economical, environmentally friendly, sustainable, and analytically efficient than previously published methods.

#### Greenness assessment

In order to achieve a high level of greenness, green chemistry focuses on eliminating all risks associated with chemical processes. Analytical processes’ beneficial environmental effects are increasingly being evaluated, and there are various ways to compare them [66]. Two greenness assessment criteria, AGREE [67], AGREEprep [68] and Complex MoGAPI [69], were used to analyze the developed approach’s eco-friendliness and greenness.

Complex MoGAPI, an improved GAPI, assesses the pre-processing procedures and the environmental friendliness of the analytical technique it employs. To evaluate the environmental friendliness of several elements, such as solvents and reagents, workup and equipment, conditions, yield, and purification, an additional hexagonal shape was added to the GAPI pictogram in Complex MoGAPI. The environmental and occupational risks of an analytical operation are assessed using the quantitative AGREE technique, which considers 12 GAC essential criteria.

AGREE is represented by a number between 0 and 1. The closer a technique is to one, the more environmentally beneficial it is. Based on ten environmental parameters, the AGREEprep^®^ Calculator metric tool prioritizes sample preparation and converts them into sub-scores on a 0–1 scale before using them to calculate the final assessment score. Each score is influenced by variables such as solvents, materials and reagents, waste generation, energy consumption, sample size, and throughput. Ethanol and BR buffer were used as green solvents to measure sunset yellow directly. As shown in Fig. S8, the method was found to be good green with a substantial AGREE score of 0.78, AGREE prep score of 0.81, and green-colored Complex MoGAPI score of 83.

This study presents the NQS Index as a comprehensive assessment of the approach’s adherence to sustainable development principles [70]. The newly developed statistic provides a thorough assessment of the method’s overall effectiveness by combining three crucial factors: environmental sustainability, analytical quality, and societal need.

The NQS Index calculation is further explained in (Table S2), which also sets down the contributions of each dimension and shows how these elements combine to create a cohesive and reliable metric that can be compared to one that has been published.

## Conclusion

This study effectively describes a novel; eco-friendly electrochemical sensor based on the electrochemical polymerization of methylene blue dye on a glassy carbon electrode for the precise quantification of SRT in pharmaceutical formulations and spiked human plasma. Using CV, DPV, and EIS, the electrochemical behavior of sertraline at PMB/GCE was examined. When compared to bare GCE, the PMB modification on the GCE significantly increased the electrochemical signal for SRT, resulting in more precise and trustworthy measurements.

The sensor demonstrated satisfactory performance in different matrices, including pharmaceutical samples and spiked plasma. The developed method allowed for the accurate quantification of SRT over a large concentration spread because it had a broad linear dynamic range (0.5–30 µM) and an extraordinarily low detection limit (0.28 µM). Overall, this work highlights the potential application of PMB/GCE in precise quantification of SRT for clinical diagnostics due to its simplicity, cost-effectiveness, high sensitivity, and potential for real-time detection compared to traditional analytical methods like HPLC or mass spectrometry. The proposed procedure showed a high degree of greenness, whiteness, and NQS assessment.

## Supplementary Information

Below is the link to the electronic supplementary material.

Supplementary Material 1

## Data Availability

The data supporting the results of this manuscript can be accessed upon request.
