# Supplementary material for: Eco-friendly and ultra-sensitive electrochemical sensor for sertraline detection in pharmaceuticals and plasma
Source: BMC Chem. 2025 Aug 12;19(1):236. doi: 10.1186/s13065-025-01602-2 (PMC12341207; doi:10.1186/s13065-025-01602-2)
Supplement: Supplementary file 1 — Supplementary Material 1 [file 13065_2025_1602_MOESM1_ESM.docx]

**Supplementary Materials**

**Eco-friendly and Ultra-sensitive Electrochemical Sensor for Sertraline Detection in Pharmaceuticals and Plasma**

**Ramy E. El-Bahnasawy^1^, Hany A. Batakoushy^1, 2^ and Hytham M. Ahmed^1, 2*^**

^1^Pharmaceutical Analysis Department, Faculty of Pharmacy, Menoufia University, Shebin Elkom, 32511, Menoufia, Egypt

^2^Department of Pharmaceutical Analytical Chemistry, Faculty of Pharmacy, Menoufia National University, 70^th^ km Cairo-Alexandria agricultural road, Menoufia, Egypt

^*^ **Corresponding Author**: **Prof. Hytham M. Ahmed** [hmaahmed@yahoo.co.uk](mailto:hmaahmed@yahoo.co.uk) (H.M.A.)

| **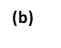** 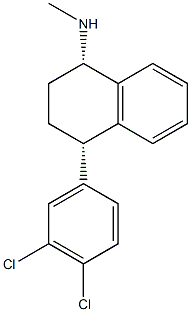  **(a)** | 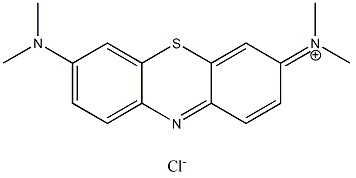 |
| --- | --- |

**Fig. S1:** The chemical structure of (a) Sertraline and (b) methylene blue


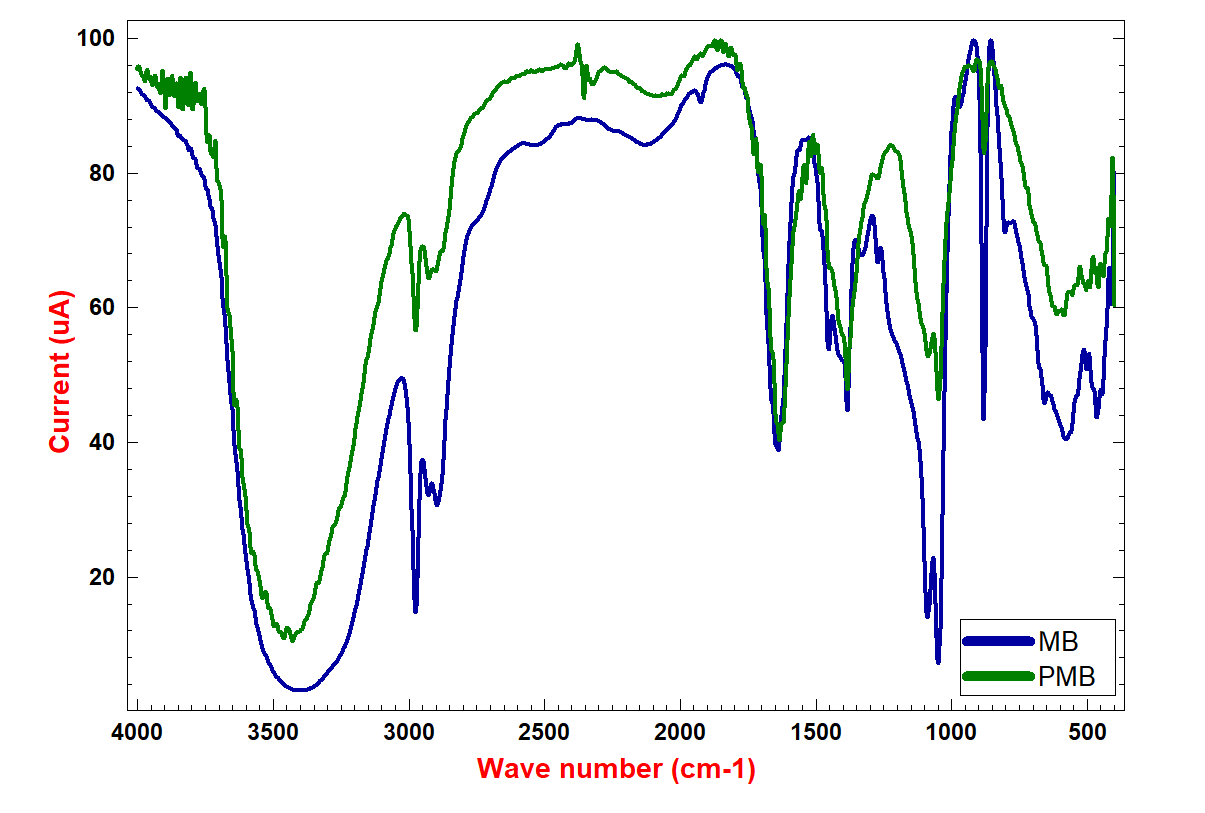


**Fig S2:** FTIR spectrum methylene blue powder and prepared polymer


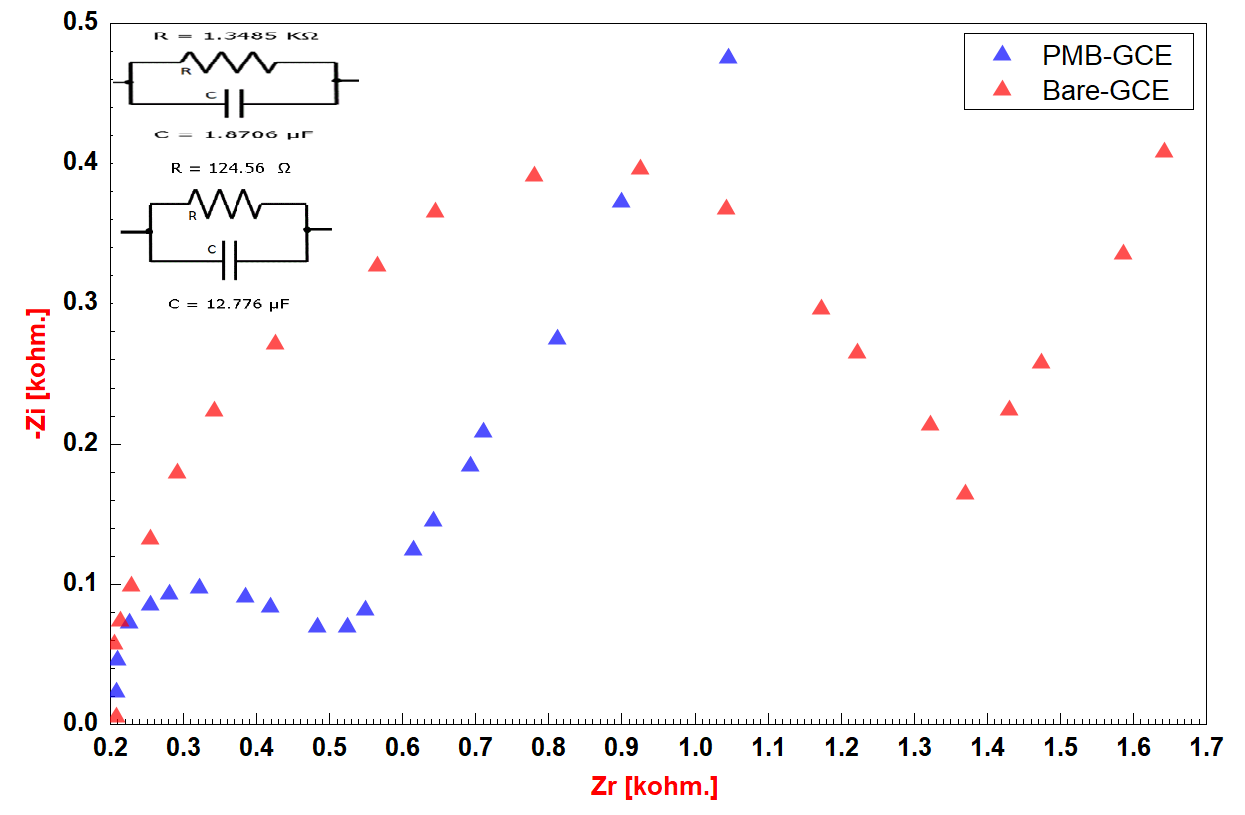


**Fig. S3:** Nyquist plot of the EIS bare GCE, PMB/GCE, and Inset of Equivalent circuit employed to fit the impedance data


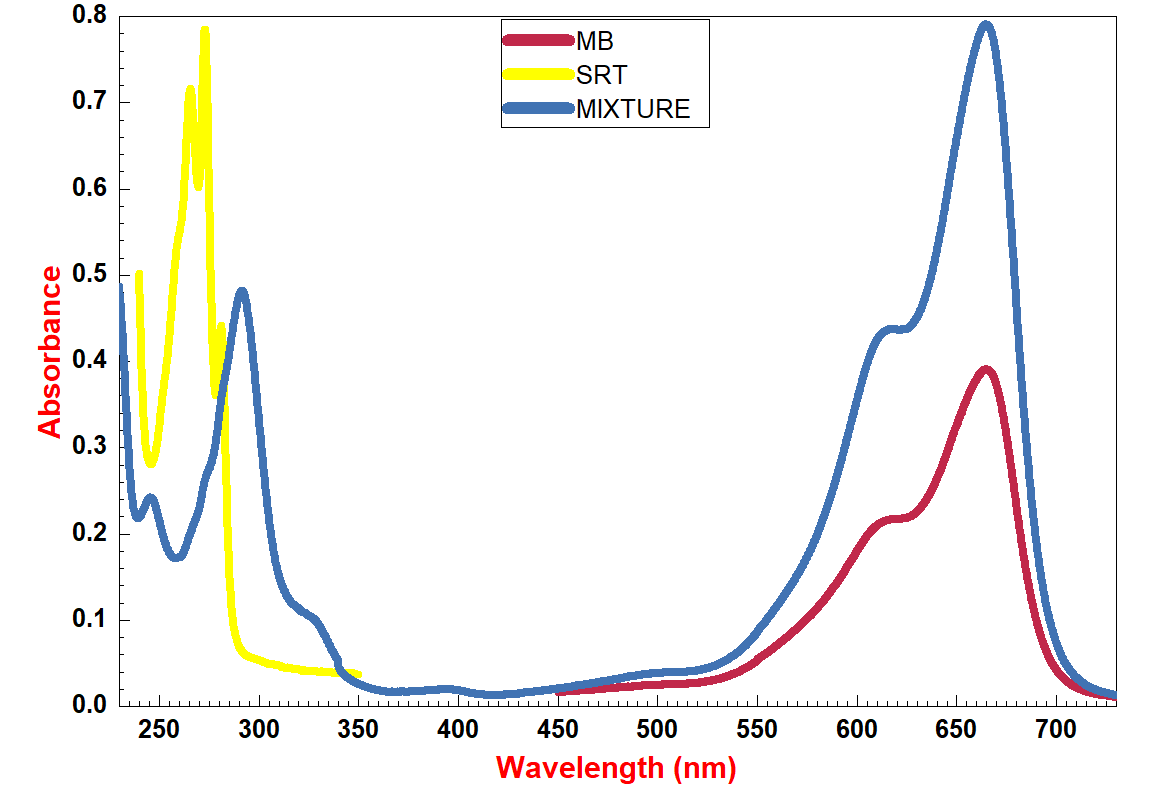
 **Fig. S4:** UV spectrum sertraline, methylene blue and mixture of sertraline and methylene blue

**
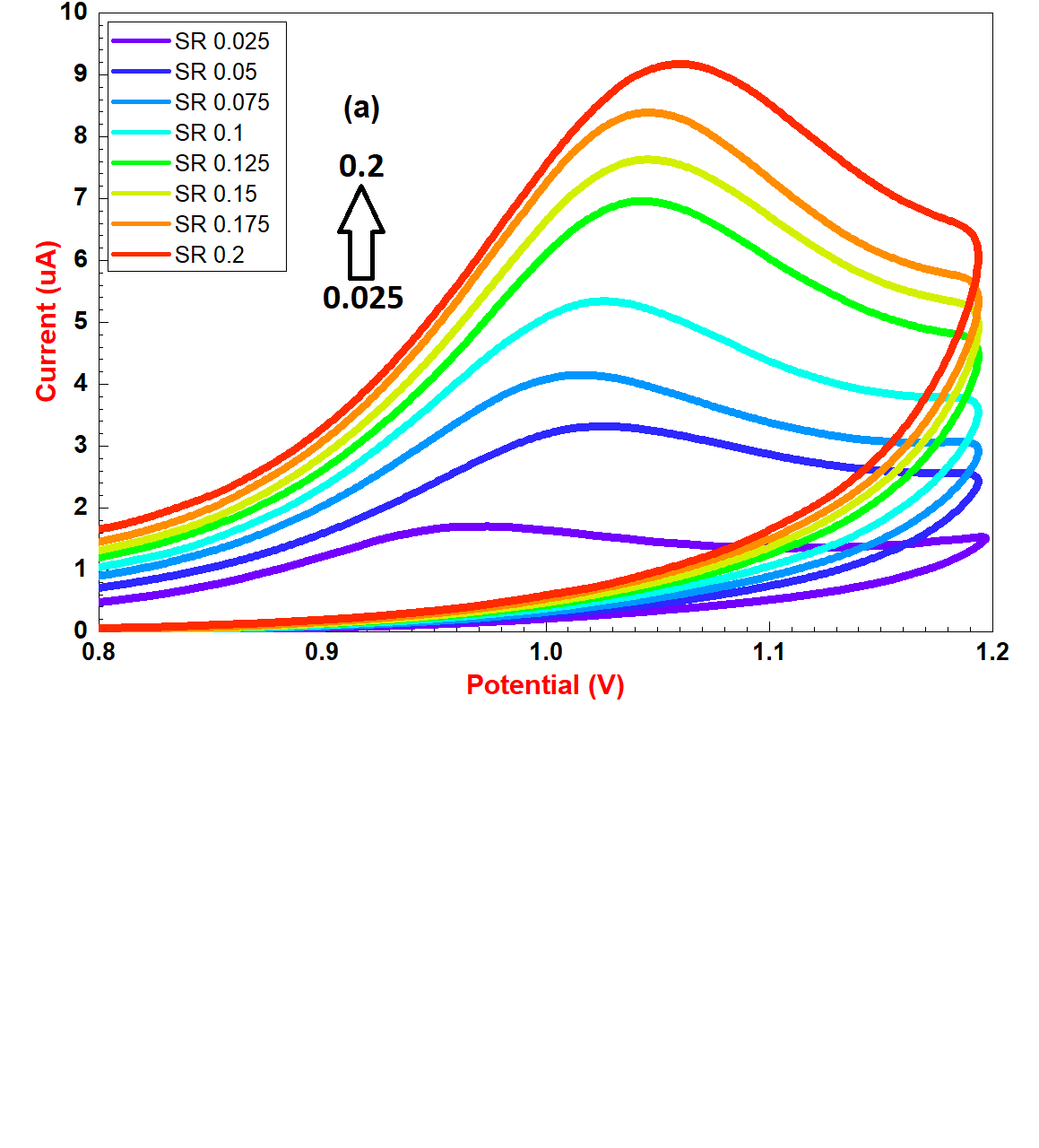
**
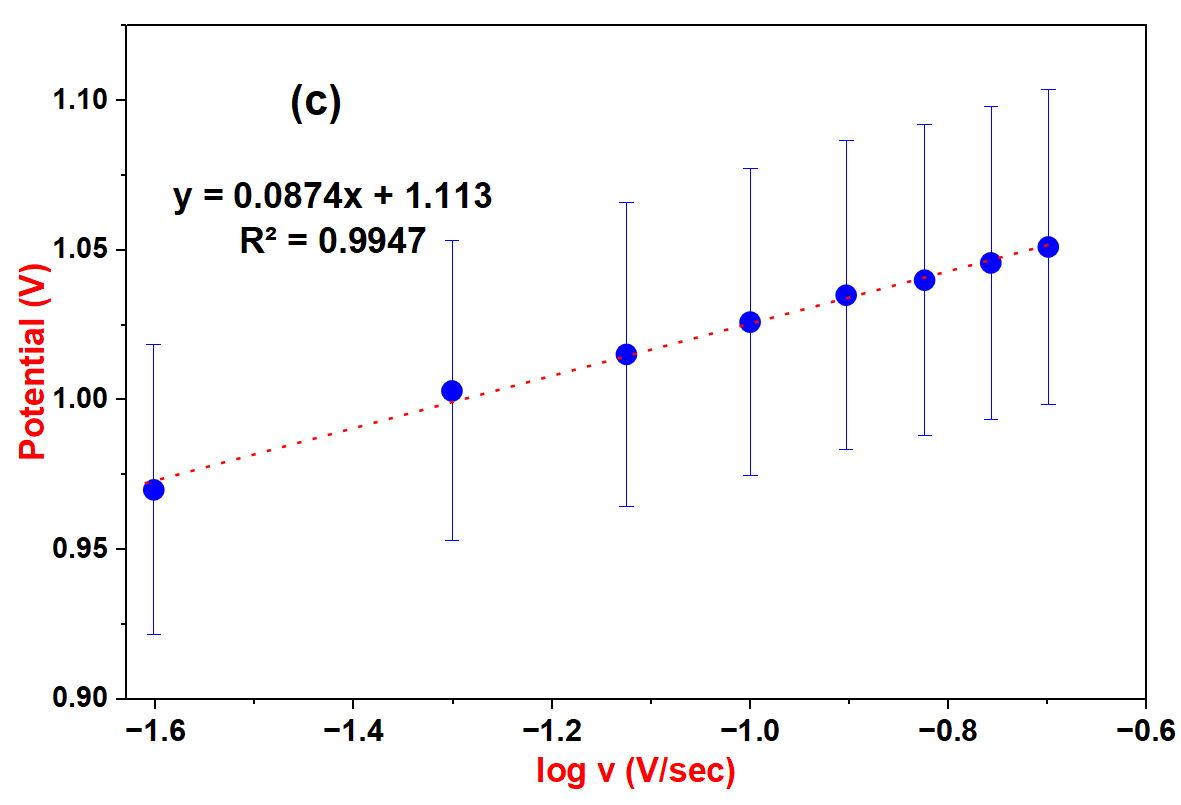

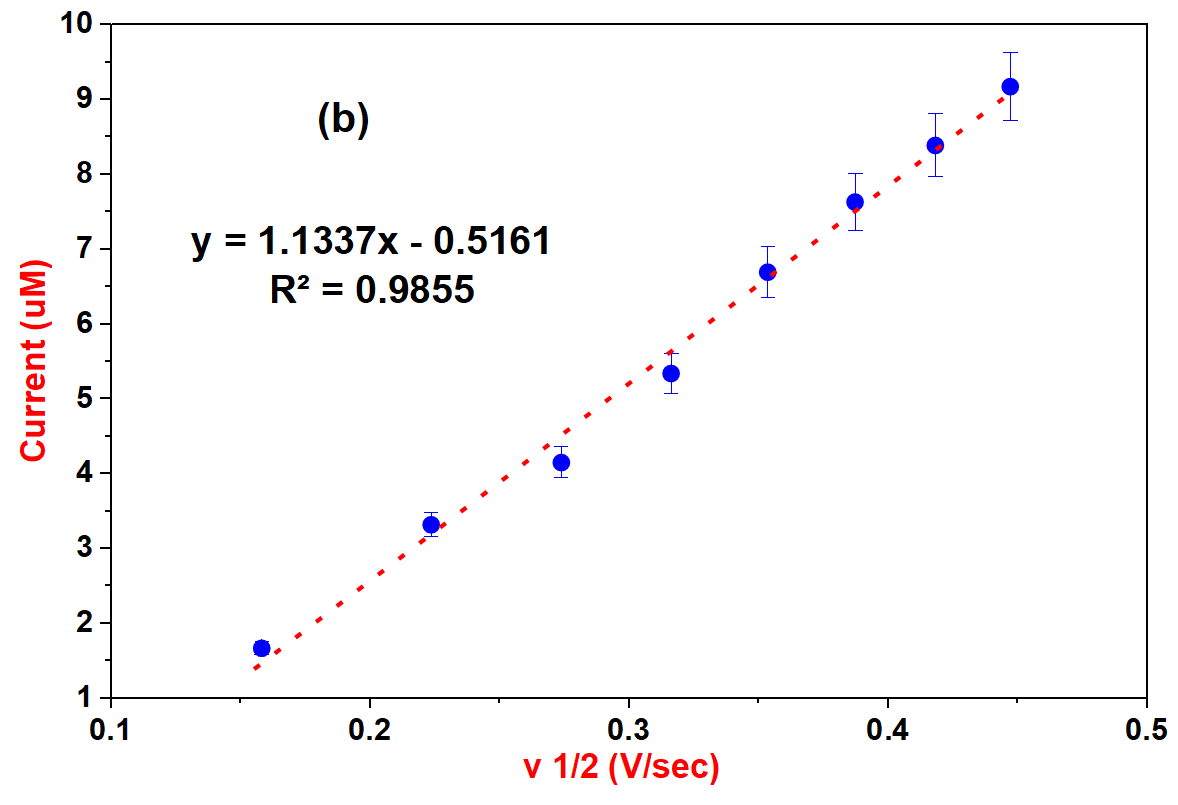


**Fig. S5:** (a) Cyclic voltammetric responses of 1.0×10^−3^ mol L^−1^ SRT at different scan rates (0.025 – 0.2 V/S) using PMB/GCE, (b) The inset displays the linear relation of Current (µA) versus square root of scan rate v ½ (V/S ) and (c) The inset displays the linear relation of Potential (mV) versus log scan rate v (V/S).


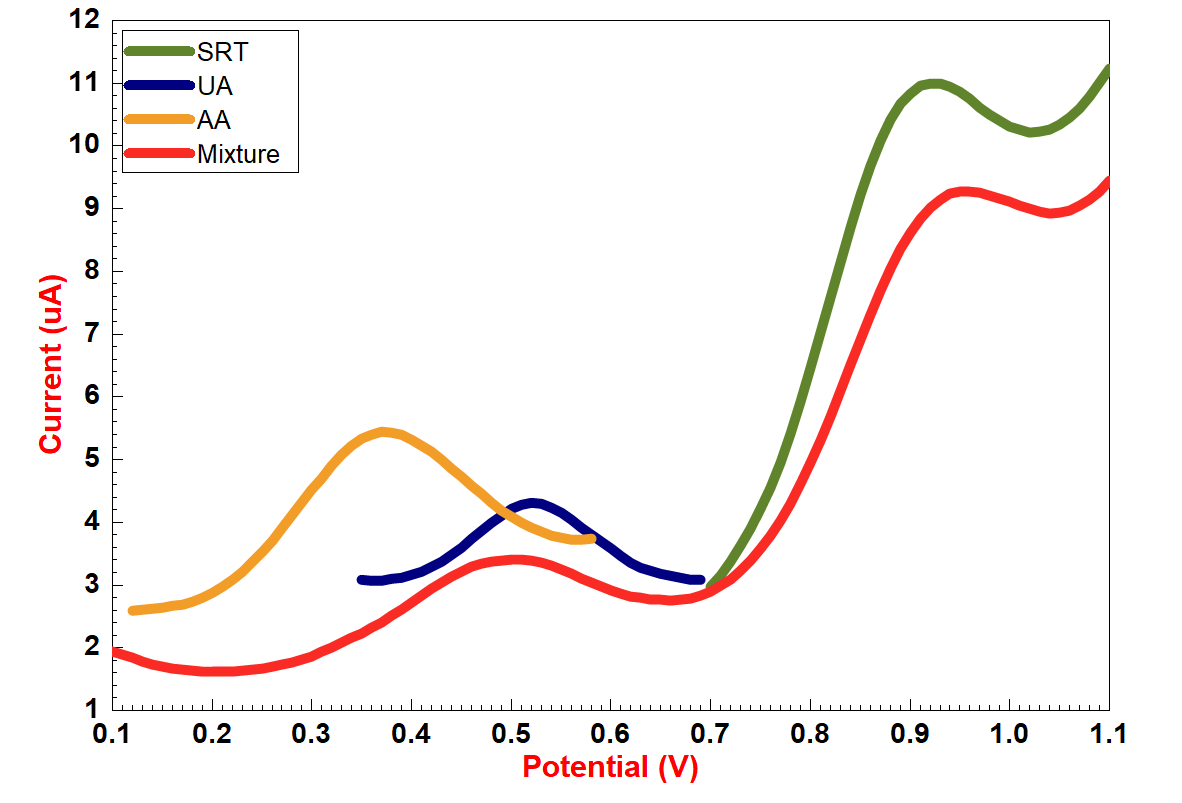


**Fig. S6:** Differential pulse voltammogram represents simultaneous determination AA (1.0 mM at pH 4.0), UA (1.0 mM at pH 4.0), SRT (0.05 mM at pH 9.0) and mixture of AA (1.0 mM), UA (1.0 mM) and SRT (0.05 mM) at pH 9.0.


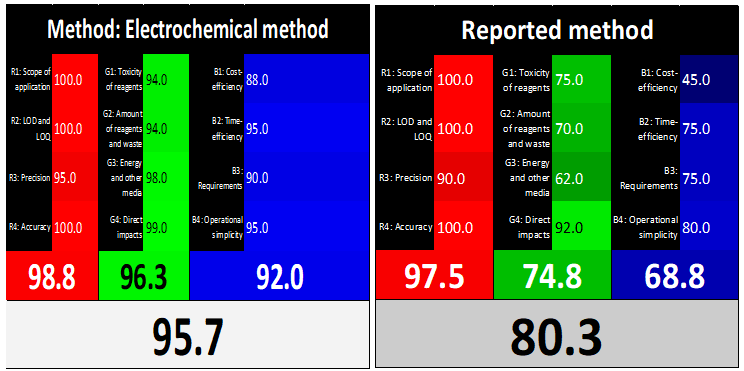


**Fig. S7:** Comparison of RGB 12 algorithm of the proposed method to the reported one


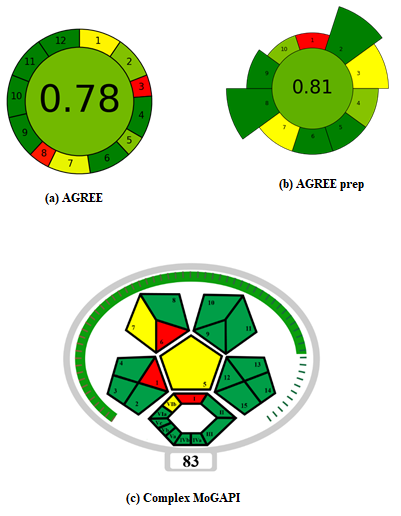


**Fig. S8:** Greenness profile assessment of the electrochemical proposed method by (a) AGREE, (b) AGREE prep, and (c) Complex MoGAPI.


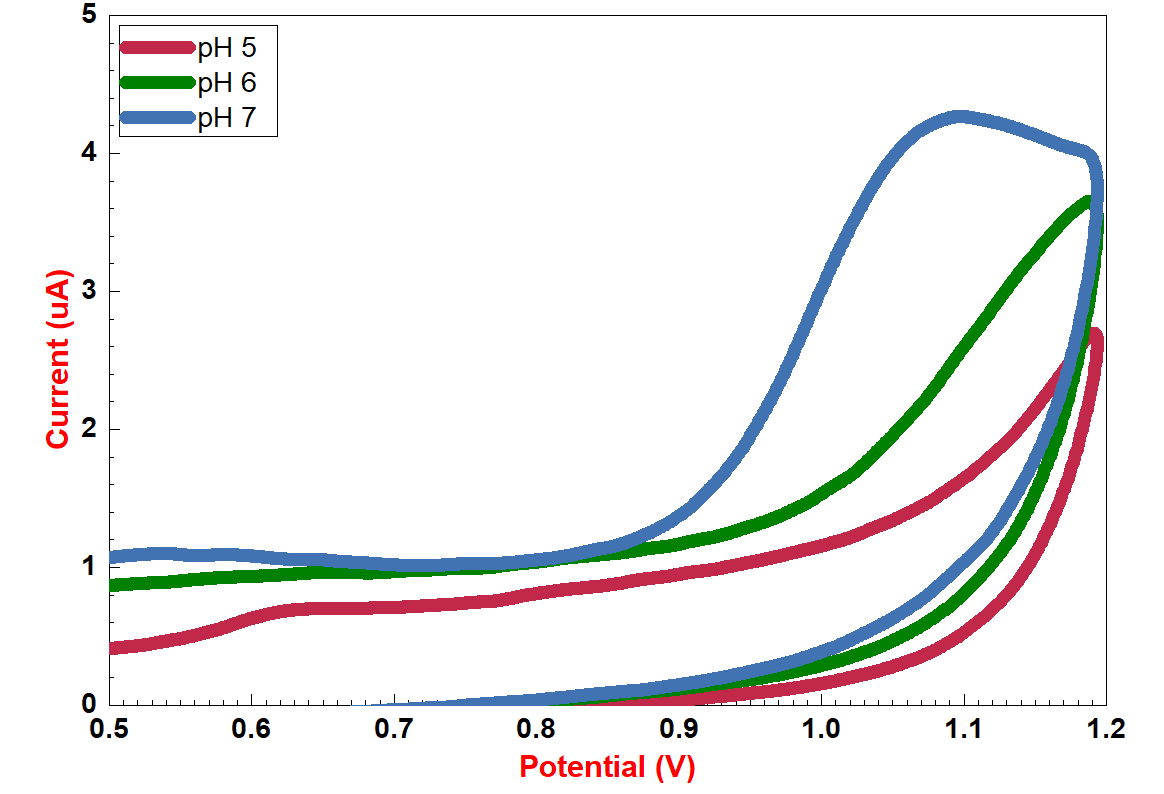


Fig. S9: Cyclic voltammetric responses of 1.0×10^−3^ mol L^−1^ SRT at pH values (5.0 – 7.0) using PMB/GCE using scan rate of 0.1 V s^−1^

**Table S1:** Comparison of some characteristics of the proposed sensor with other reported ones for SRT determination

| **Electrode** | **Technique** | **Linearity range (M)** | **Detection limit LOD (M)** | **Real sample** | **Reference** |
| --- | --- | --- | --- | --- | --- |
| GCE^a^ | OSWV^b^ | 4.00 × 10^−5^ – 8.00 × 10^−4^ | 1.04 × 10^−5^ | Tablets | [15] |
| R/GCE^c^ | DPV^d^ | 3.00 × 10^−6^ – 9.00 × 10^−5^ | 1.00 × 10^−6^ | Tablets | [16] |
| PVC membrane  CMCP sensor^e^ | Potentiometry | 1.00 × 10^−5^ – 1.00 × 10^−2^ | 2.80 × 10^−6^  9.55 × 10^−6^ | Pure raw material, Tablets | [17] |
| MIP membrane^f^ | Potentiometry | 1.00 × 10^−6^ – 1.00 × 10^−2^ | 8.00 × 10^−7^ | Tablets, urine, serum | [18] |
| PMB/GCE | DPV | 0.5 x 10^-6^ – 30.00 x 10^-6^ | 2.80 x 10^-7^ | Tablets, plasma samples | This Work |

^a^ Glassy carbon electrode, ^b^ Osteryoung Square Wave Voltammetry, ^c^ Rutin-modified glassy carbon electrode, ^d^ Differential pulse voltammetry, ^e^ chemically modified carbon paste (CMCP) sensor, ^f^ Molecularly imprinted polymer membrane
M Molarity

**Table S2:** Need, Quality, and Sustainability (NQS) Index calculations

|  | Proposed | Reported |
| --- | --- | --- |
| Need | 100 | 100 |
| Quality | 94 | 92 |
| Sustainability | 60 | 35 |
| **NQS Index** | **85** | **76** |

**
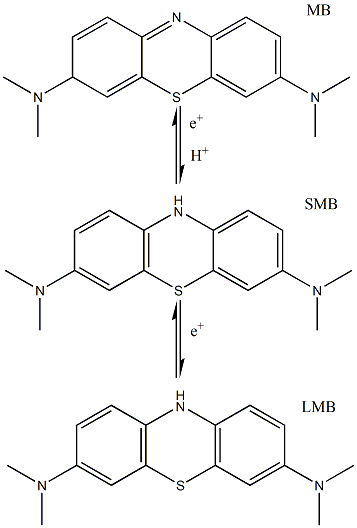
**

**Scheme S1:** The suggested oxidation mechanism of methylene blue monomer

**
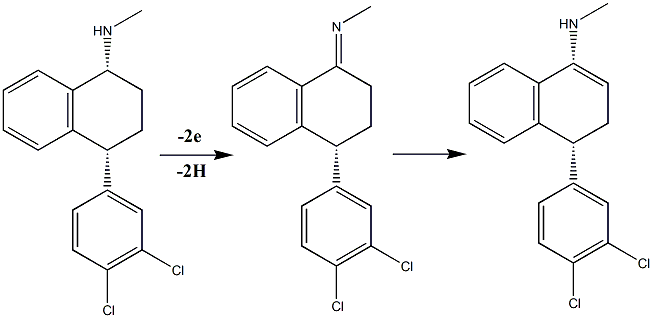
**

**Scheme S2:** The suggested oxidation mechanism of sertraline
